# Supplementary material for: Distinct gene regulatory dynamics drive skeletogenic cell fate convergence during vertebrate embryogenesis
Source: Nat Commun. 2025 Mar 4;16:2187. doi: 10.1038/s41467-025-57480-8 (PMC11880379; doi:10.1038/s41467-025-57480-8)
Supplement: Supplementary file 1 — Supplementary Information [file 41467_2025_57480_MOESM1_ESM.pdf]

SUPPLEMENTARY INFORMATION

Distinct Gene Regulatory Dynamics Drive Skeletogenic Cell Fate Convergence During Vertebrate Embryogenesis

Menghan Wang[1,3,#], Ana Di Pietro-Torres[1,#], Christian Feregrino[1,4], Maëva Luxey[1,5], Chloé Moreau[1], Sabrina Fischer[1], Antoine Fages[1], Danilo Ritz[2], Patrick Tschopp[1,*]

[1]Zoology, Department of Environmental Sciences, University of Basel, 4051 Basel, Switzerland
[2]Proteomics Core Facility, Biozentrum, University of Basel, 4051 Basel, Switzerland
[3]Present address: Department of Biomedicine, University of Basel Children's Hospital, 4031 Basel, Switzerland
[4]Present address: Max Planck Institute for Molecular Genetics, 14195 Berlin, Germany
[5]Present address: MeLis, CNRS UMR 5284, INSERM U1314, Université Claude Bernard Lyon 1, Institut NeuroMyoGène, 69008 Lyon, France
[#]These authors contributed equally: Menghan Wang, Ana Di Pietro-Torres.

[*]Correspondence Patrick Tschopp
Tel.: +41 61 207 56 49
patrick.tschopp@unibas.ch

The PDF file includes:
Supplementary Figures 1 to 9

Other Supplementary Materials for this manuscript include:
Supplementary Data 1 [Mass spectrometry sample preparation and experimental procedures, differential abundance tests results]

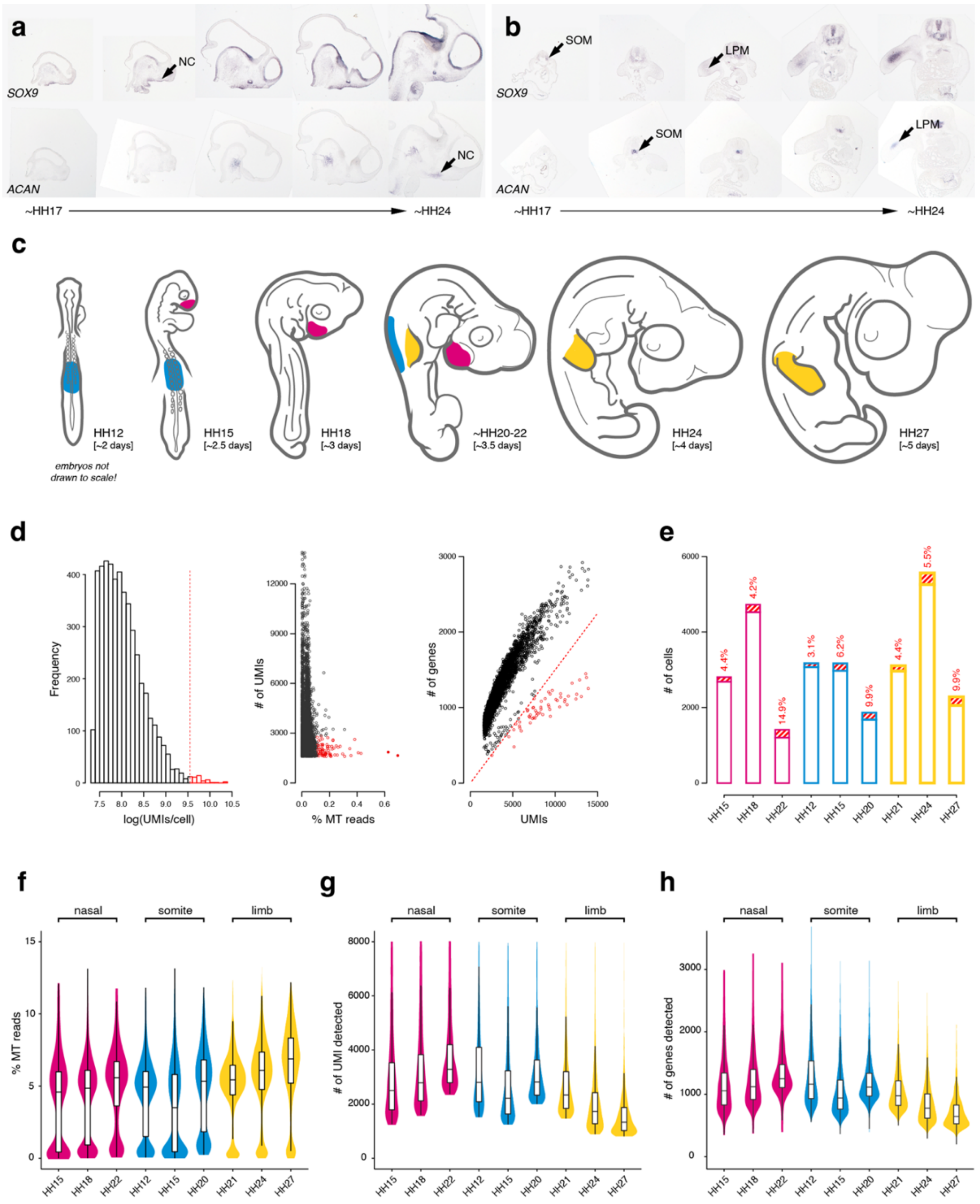

Supplementary Figure 1. ***In situ*** **dynamics of skeletogenic induction across three embryonic lineages and sampling scheme and quality metrics of single-cell transcriptomics experiments. a**, **b** Chromogenic *in situ* hybridizations against *SOX9* and *ACAN* on cranial sagittal (**a**) and brachial transversal (**b**) cryosections spanning embryonic stages HH17 to HH24. First appearance of signal in the respective embryonic lineages is marked by an arrow (NC: neural crest, SOM: somite, LPM: lateral plate mesoderm). **c** Schematic drawings of the embryonic stages and tissues sampled for single-cell transcriptomics experiments. Tissues are color-coded according to the embryonic lineage their prospective skeletogenic cells derive from (neural crest (magenta), somitic mesoderm (blue) lateral plate mesoderm (yellow)) and embryonic stages are indicated according to Hamburger-Hamilton (HH, approximate days of incubation indicated in brackets). **d** Representative quality control (QC) plots (sample 'HH18', nasal) to exclude low quality cells (in red) based on overall high UMI counts (left), high relative mitochondrial counts (middle) and low gene detection-per-UMI rate (right). **e** Total cell numbers of single-cell transcriptomes obtained for each sample, with cells removed during QC indicated in red. Between 3.1% to 14.9% of the cells were removed, based on the criteria shown in (**d**). **f-h** Violin plots of percent mitochondrial reads (**f**), number of UMIs (**g**) and number of genes detected (**h**), in cells of the nine scRNA-seq samples after QC, color-coded by origin. Center line of boxplots = median, box limits = quartiles, and whiskers = 1.5 x inter quartile range.

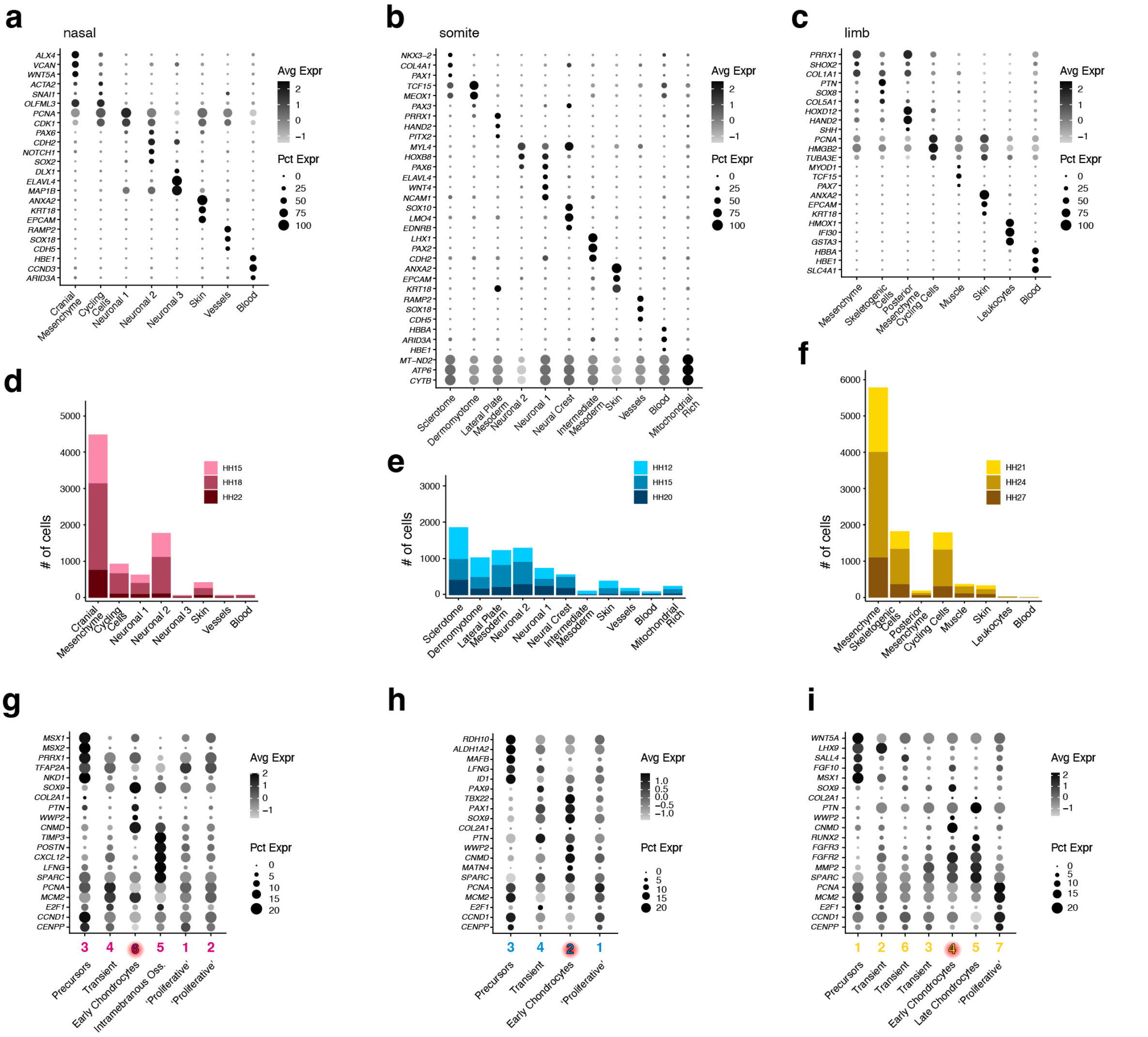

Supplementary Figure 2. 'Broad' and 'fine' cell cluster identification in scRNA-seq datasets. a-c Dot plots of select marker genes expression used for 'broad' cell cluster annotations. **d-f** Stacked bar plots indicating stage-wise contributions to the total number of cells in 'broad' clusters, color coded by anatomical origin and embryonic stage. **g-i** Dot plots of select marker genes expression used for 'fine' cell cluster annotations.

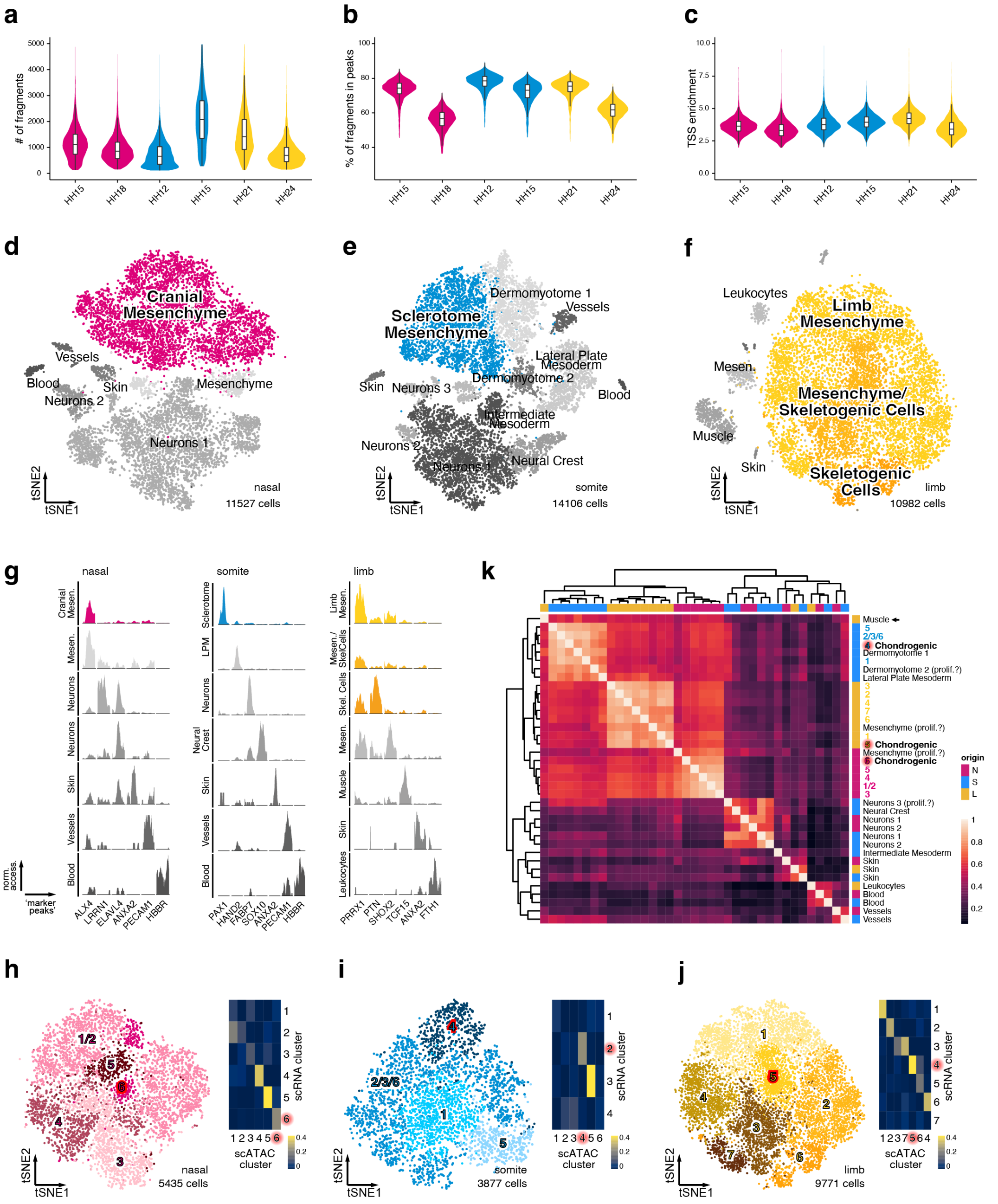

Supplementary Figure 3. The single-cell chromatin accessibility landscape of skeletogenic induction across three embryonic lineages. **a**-**c** Violin plots of quality control metrics showing number of fragments detected (**a**), percentage of fragments in peaks (**b**) and transcription start site (TSS) enrichment (**c**), in cells of the six scATAC-seq samples color-coded by origin. Center line of boxplots = median, box limits = quartiles, and whiskers = 1.5 x inter quartile range. **d**-**f** tSNE representations of the three scATAC-seq datasets and ‘broad’ cell type annotations, with mesenchymal populations used for ‘fine’ re-clustering highlighted in color. **g** Pseudobulk aggregate genome accessibility tracks of peaks near ‘broad’ cell type-specific marker genes. **h**-**j** tSNE representations of re-clustered mesenchymal cells of nasal (**h**), somite (**i**) and limb (**j**) origins, with ‘fine’ cluster annotations and normalized scores based on non-negative least squares (NNLS) regression, between gene activity from ATAC clusters and gene expression from RNA clusters. High values are indicative of cell type correspondences between scATAC and scRNA clusters. Early chondrogenic clusters are highlighted in red. **k** Unsupervised hierarchical clustering and heatmap representation of pairwise Spearman’s rank correlation coefficients of differentially accessible peaks in pseudobulk chromatin accessibility data of ‘broad’ and ‘fine’ mesenchymal clusters. Anatomical origins of pseudobulks are indicated by color code. All mesenchymal pseudobulks cluster by embryonic origins, including early chondrogenic cells (highlighted in red) and somite-derived muscles in the limb sample (arrow).

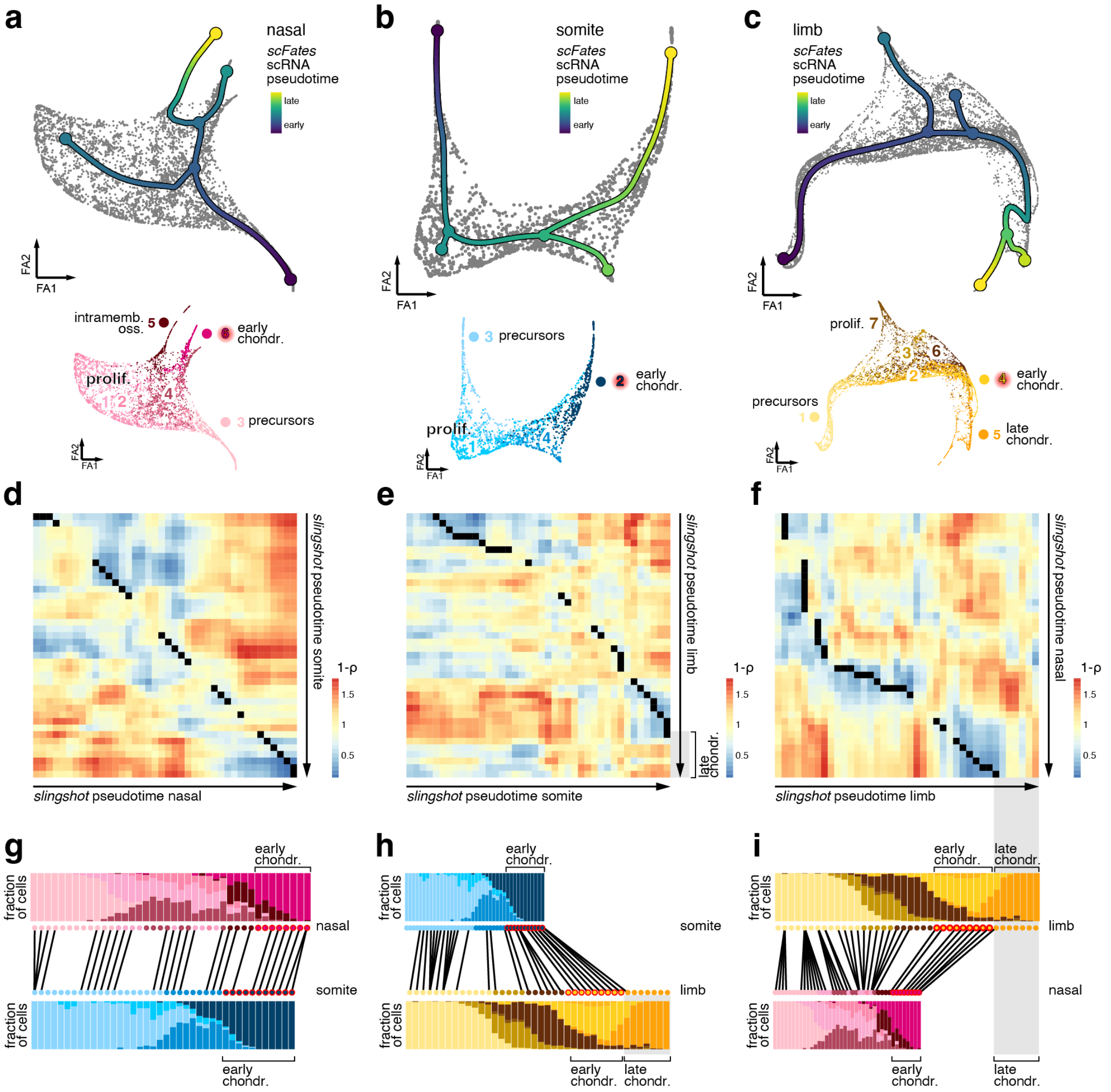

Supplementary Figure 4. Skeletogenic convergence across three embryonic lineages. **a-c** *scFates* pseudotime trajectories visualized on *ForceAtlas2* layouts, for mesenchymal 'fine' clusters of nasal (**a**), somite (**b**) and limb origin (**c**). **d-i** *TrAGEDy* alignments of embryonic origin-specific chondrogenic *slingshot* pseudotime trajectories. **d-f** Pairwise expression dissimilarities of the chondrogenic module 'IMM' were calculated by Spearman correlation across all pseudotimes and embryonic origins, and are displayed as 1-ρ. Dynamic time warping identifies skeletogenic convergence across all three embryonic lineages, except for the 'late chondrocytes' population in the limb sample that is not accounted for in our nasal and somite data. **g-i** *TrAGEDy* alignment plots of interpolated points of chondrogenesis trajectories and cell type contributions per pseudotime bin, displayed as stacked bar plots. Pseudotime bins where most cells are classified as 'early chondrogenic' are highlighted in red.

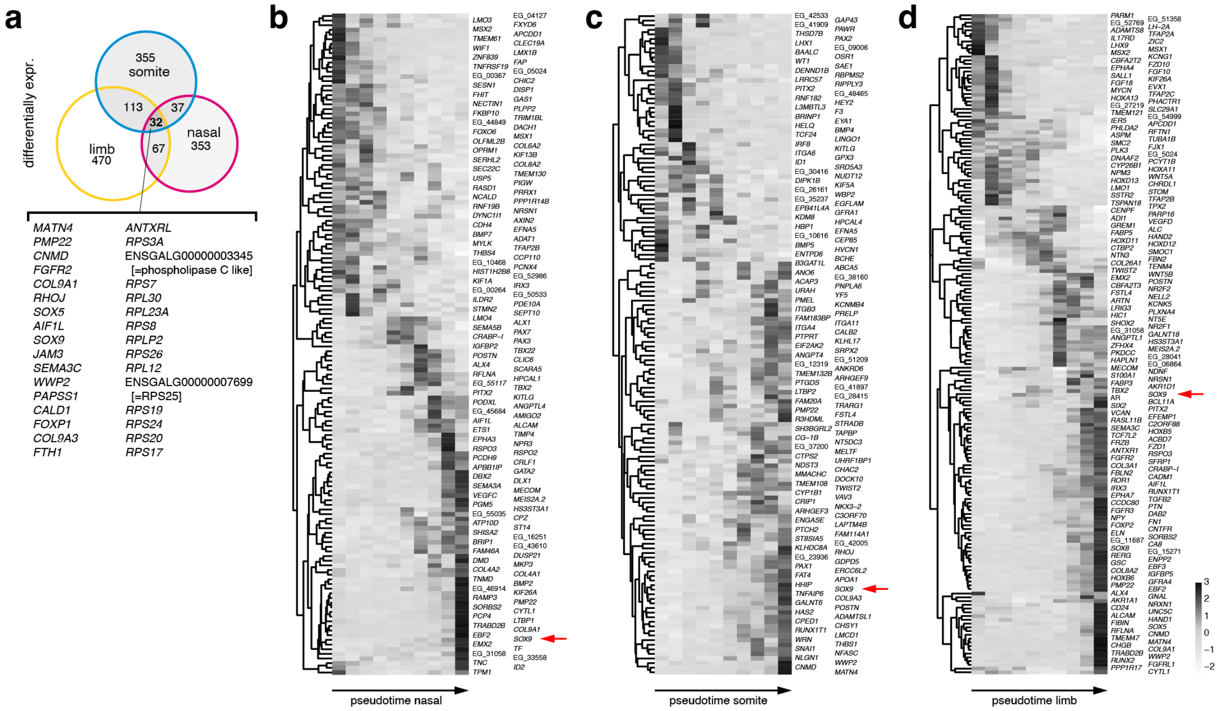

Supplementary Figure 5. Core skeletogenic genes and embryonic origin-specific transcriptional dynamics. **a** Venn diagram displaying the overlap of differentially expressed genes in chondrogenic cells compared to other mesenchymal populations of the three embryonic origins. **b-d** Z-score scaled RNA levels of dynamically expressed genes along the chondrogenic trajectories of nasal (**b**), somite (**c**) and limb (**d**) mesenchymal and chondrogenic cells. *SOX9* expression is highlighted by a red arrow. Genes without annotated symbol are labeled with 'EG_', followed by the 5 last digits of their ENSGAL identifiers.

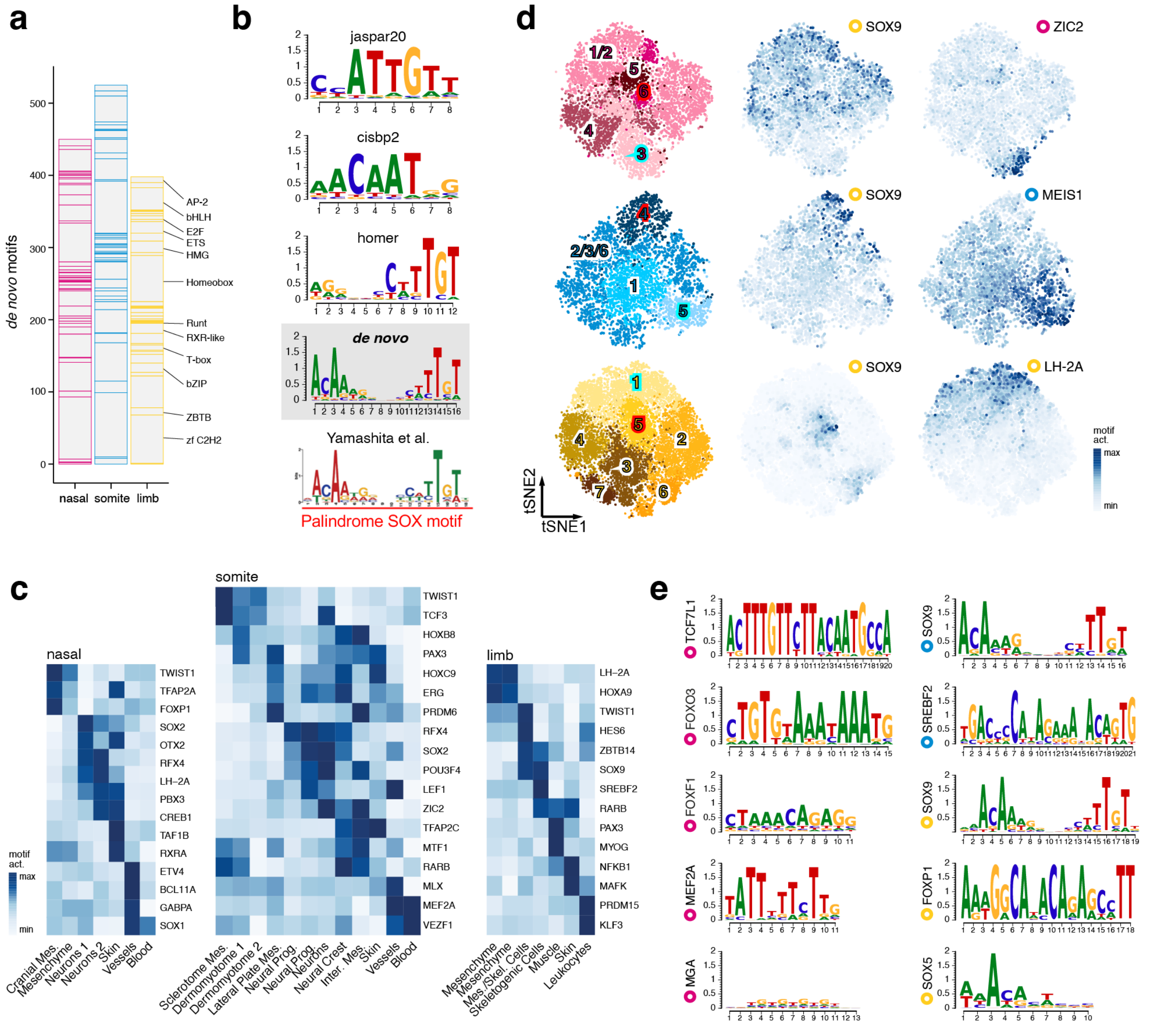

Supplementary Figure 6. *De novo* identification and annotation of transcription factor binding motifs. **a** Transcription factor (TF) family distribution of all *de novo* identified binding motifs in all populations across the three embryonic origins. **b** Publicly available position weight matrices for SOX9 in 'jaspar20', 'cisbp2' and 'homer' databases. Our *de novo* position weight matrix resembles more closely the one identified using SOX9 ChIP-seq data in chicken embryonic limbs*. **c** Differential 'broad' cluster-specific motif activities across embryonic origins. **d** tSNE representations of re-clustered mesenchymal cells, with motif activity heatmaps plotted for a core skeletogenic factor (SOX9, middle) and an embryonic origin-specific precursor factor (ZIC2, MEIS1, LH-2A, right). 'Early chondrogenic' clusters are highlighted in red, 'mesenchymal' clusters in turquoise (left). **e** Position weight matrices of the ten commonly chondrocyte-enriched TF motifs across embryonic origins. The embryonic origin in which the respective motif was identified is indicated by color-coded circles.

* SOX9 ChIP-seq position weight matrix from Yamashita et al.[36], re-used under CC-BY 4.0 license

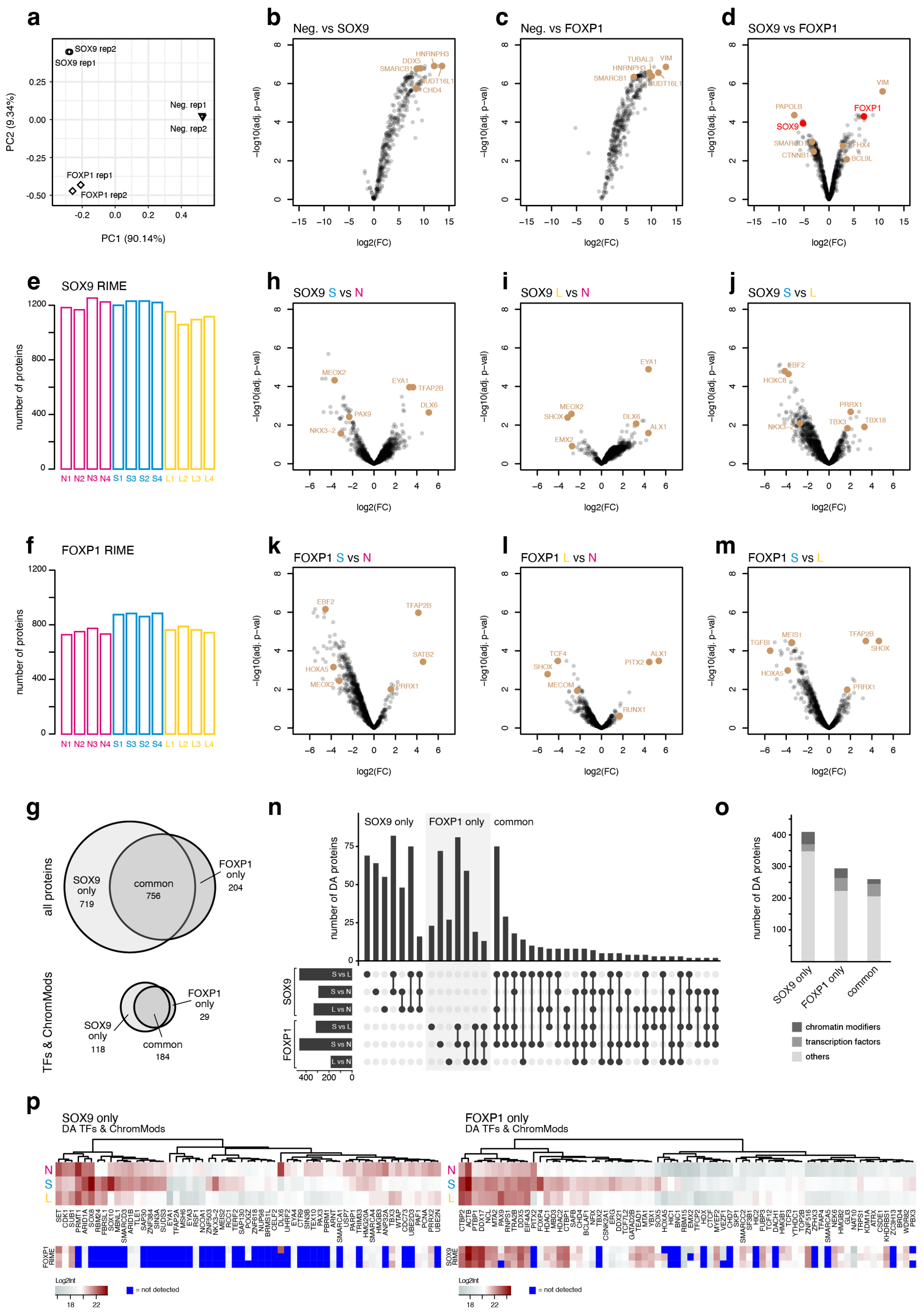

Supplementary Figure 7. Identification of lineage-specific protein interaction profiles for SOX9 and FOXP1 using Rapid Immunoprecipitation Mass spectrometry of Endogenous proteins (RIME). a Principal component analysis of replicate RIME test runs in limb tissue using antibodies against SOX9, FOXP1, IgG (Neg. rep1) or beads only (Neg. rep2). Numbers in brackets indicate the percentage of total variance explained by PC1 and PC2. **b-d** Volcano plots of differentially abundant proteins in limb RIME experiments, contrasting negative control and SOX9 immunoprecipitation (**b**), negative control and FOXP1 immunoprecipitation (**c**), and SOX9 and FOXP1 immunoprecipitation (**d**).

Select enriched proteins are highlighted, with the two targeted transcription factors colored red in (**d**). **e**, **f** Total number of proteins detected in lineage-specific quadruplicate SOX9 (**e**) and FOXP1 (**f**) RIME experiments, in neural crest- (N, magenta), somitic mesoderm- (S, blue) and lateral plate mesoderm-derived (L, yellow) tissues. **g** Overlap of identified overall proteins (top) and transcription factors (TFs) and chromatin modifiers (ChromMods) (bottom), in SOX9 and FOXP1 RIME experiments. **h**-**j** Volcano plots of differentially abundant proteins in SOX9 RIME experiments, contrasting somite- and neural crest-derived tissues (**h**), limb- and neural crest-derived tissues (**i**), and somite- and limb-derived tissues (**j**). **k**-**m** Volcano plots of differentially abundant proteins in FOXP1 RIME experiments, contrasting somite- and neural crest-derived tissues (**k**), limb- and neural crest-derived tissues (**l**), and somite- and limb-derived tissues (**m**). **n** *UpSet* plot of significantly differentially abundant (DA) proteins, identified pairwise across embryonic origins for SOX9 and FOXP1 RIME experiments (see **h**-**m**). **o** Stacked barplots indicating the number of significantly differentially abundant proteins specific to SOX9 or FOXP1 RIME experiments, or common (shared) between the two. Numbers of transcription factors and chromatin modifiers are indicated by grey shading. **p** Fully annotated heatmap representations of averaged log2-normalized intensities for SOX9-specific (left) and FOXP1-specific (right) differentially abundant transcription factors and chromatin modifiers, across embryonic origins (see Fig. 4m, main text). The relative enrichment (=heatmap) or complete absence (=blue) of the corresponding proteins in the RIME experiments targeting the other candidate factor are indicated below.

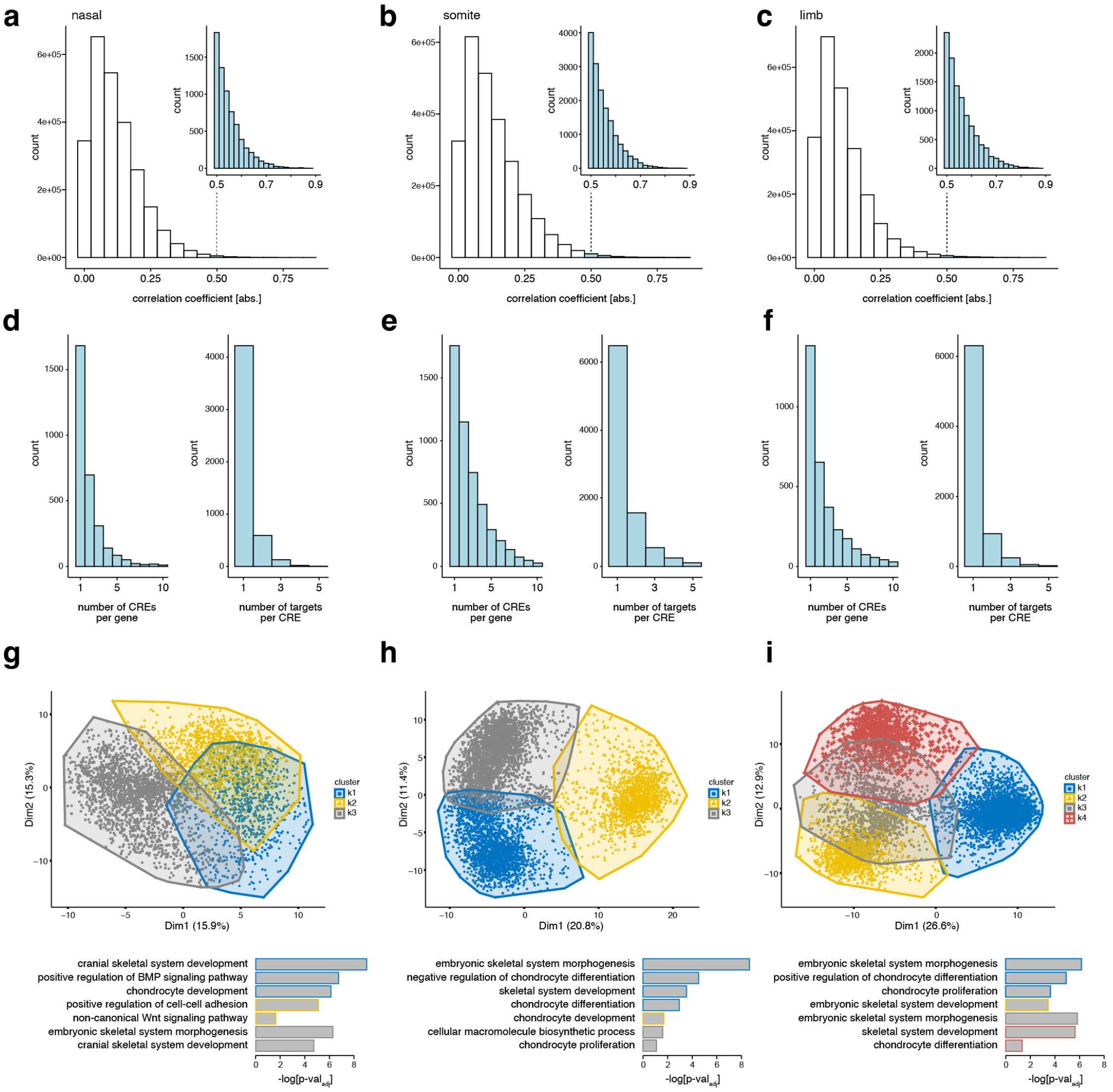

Supplementary Figure 8. Identification and metrics of predicted peak-to-gene links from single-cell functional genomics data. **a-c** Histograms of absolute correlation coefficients of identified peak-to-gene links. Only peak-to-gene links with an absolute correlation coefficient larger than 0.5 were considered for further analysis (inset). **d-f** Peak-to-gene links statistics. The majority of identified peak-to-gene links predicted 1-3 *cis*-regulatory elements (CRE) per target gene, and most CREs to contact only one target gene. **g-i** Cluster plots of hkmeans clustering based on peak accessibility of peak-to-gene links. Functional enrichment analyses results are displayed below, with clusters-of-origin indicated by colored frame.

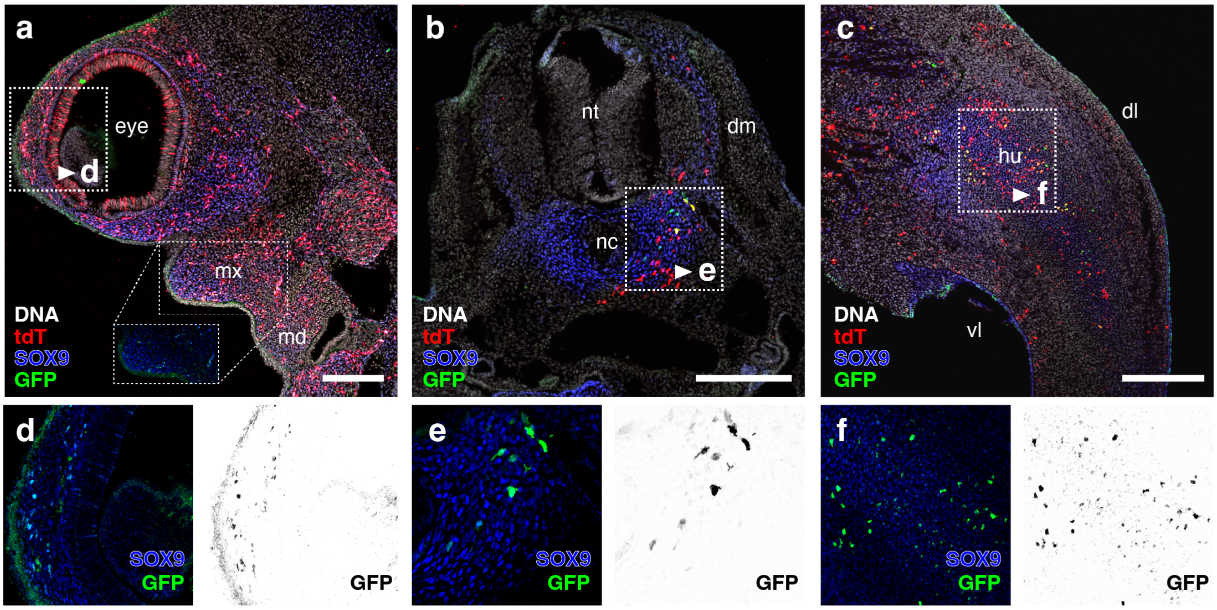

Supplementary Figure 9. Chondrocyte-restricted activities of lineage-specific enhancer reporter constructs. **a-f** Despite wide-spread electroporation, as evidenced by a co-electroporation marker (=tdT-positive), enhancer reporter activity (=GFP-positive) is restricted to cells of early chondrogenic condensations (=SOX9-positive) (see also insets in (**d-f**) for details), in the neural crest (**a**), somitic (**b**) and lateral plate mesoderm lineages (**c**). Scale bars in (**a-c**): 200mm. mx: maxilla, md: mandible, nt: neural tube, dm: dermomyotome, hu: humerus, dl: dorsal, vl: ventral.
