# Supplementary material for: Distinct gene regulatory dynamics drive skeletogenic cell fate convergence during vertebrate embryogenesis
Source: Nat Commun. 2025 Mar 4;16:2187. doi: 10.1038/s41467-025-57480-8 (PMC11880379; doi:10.1038/s41467-025-57480-8)
Supplement: Supplementary file 2 — Description of Additional Supplementary Files [file 41467_2025_57480_MOESM2_ESM.pdf]

Description of Additional Supplementary Files

Supplementary Data 1- Mass spectrometry sample preparation and experimental procedures, differential abundance tests results
