# Supplementary material for: Distinct gene regulatory dynamics drive skeletogenic cell fate convergence during vertebrate embryogenesis
Source: Nat Commun. 2025 Mar 4;16:2187. doi: 10.1038/s41467-025-57480-8 (PMC11880379; doi:10.1038/s41467-025-57480-8)
Supplement: Supplementary file 4 — Reporting Summary [file 41467_2025_57480_MOESM4_ESM.pdf]

nature portfolio

Corresponding author(s): Patrick Tschopp [patrick.tschopp@unibas.ch]

Last updated by author(s): 24.1.2025

Reporting Summary

Nature Portfolio wishes to improve the reproducibility of the work that we publish. This form provides structure for consistency and transparency in reporting. For further information on Nature Portfolio policies, see our Editorial Policies and the Editorial Policy Checklist.

Statistics

For all statistical analyses, confirm that the following items are present in the figure legend, table legend, main text, or Methods section.

n/a	Confirmed	
☐	☒	The exact sample size (*n*) for each experimental group/condition, given as a discrete number and unit of measurement
☐	☒	A statement on whether measurements were taken from distinct samples or whether the same sample was measured repeatedly
☐	☒	The statistical test(s) used AND whether they are one- or two-sided<br>*Only common tests should be described solely by name; describe more complex techniques in the Methods section.*
☒	☐	A description of all covariates tested
☐	☒	A description of any assumptions or corrections, such as tests of normality and adjustment for multiple comparisons
☐	☒	A full description of the statistical parameters including central tendency (e.g. means) or other basic estimates (e.g. regression coefficient) AND variation (e.g. standard deviation) or associated estimates of uncertainty (e.g. confidence intervals)
☐	☒	For null hypothesis testing, the test statistic (e.g. *F*, *t*, *r*) with confidence intervals, effect sizes, degrees of freedom and *P* value noted<br>*Give P values as exact values whenever suitable.*
☒	☐	For Bayesian analysis, information on the choice of priors and Markov chain Monte Carlo settings
☒	☐	For hierarchical and complex designs, identification of the appropriate level for tests and full reporting of outcomes
☒	☐	Estimates of effect sizes (e.g. Cohen's *d*, Pearson's *r*), indicating how they were calculated

Our web collection on statistics for biologists contains articles on many of the points above.

Software and code

Policy information about availability of computer code

Data collection

(1) For read processing and quantification of functional genomics data, we used CellRanger v2.0.1 (10x Genomics) for scRNA-seq and CellRanger ATAC v1.2.0 (10x Genomics) for scATAC-seq(PEAK_MERGE_DISTANCE was changed to 50, with all other parameters at default settings), using our in-house improved GRCg6a genome annotation with elongated 3' UTRs.
(2) For proteomic data, the acquired raw-files were searched using MSFragger (v. 4.1) implemented in FragPipe (v. 22.0) against a Gallus gallus database (consisting of 43711 protein sequences downloaded from Uniprot on 20231218) and 392 commonly observed contaminants using the "LFQ-MBR" workflow.

Data analysis

We used previously published computational tools, with specific settings detailed under https://github.com/wangmhan/skeletoConvergence
(1) scRNA-seq Data Normalization, Dimensionality Reduction and Clustering
Using the R package Seurat (v4), UMI counts were normalized by sequencing depth and log transformed. A cell cycle score was calculated using SCRAN. Variations of sequencing depth, mitochondrial UMI percentage, and the difference in S and G2M cycle scores were regressed out, using SCTransform from Seurat. Genes with a higher value of standardized variance than the sum of median and median absolute deviation were considered as 'highly variable'. These steps were carried out independently for the three stages of the three embryonic origins. Using Seurat, we integrated samples from the same embryonic origin and used principal component analysis (PCA) on highly variable genes, followed by tSNE and FFT-accelerated Interpolation-based tSNE algorithms for non-linear dimensionality reduction on the first 19 (nasal), 21 (somite) and 19 (limb) principal components. Using Seurat functions, we performed Leiden graph-based clustering on all cells with a resolution of 0.2 (='broad clustering'). A second round of clustering was conducted on select mesenchyme populations, with resolutions of 0.4 (somite, limb) and 0.5 (nasal) (='fine clustering'). Cell type assignments of clusters were based on visual inspection of known marker gene expression patterns, and the activity of the two previously identified early chondrogenic gene expression modules 'IMM' and 'RED' using the Seurat function 'AddModuleScore'.
(2) Differential Expression Analysis

Differential expression analysis was based on a logistic regression framework using Seurat, with cell cycle differences and embryonic stages as latent variables. Genes expressed in at least 10% of the cells and showing differences with an adjusted p-value <0.05 and a log fold change >0.5 ('broad') or >0.25 ('fine') were considered as significantly differentially expressed. To minimize batch effects, differential expression analysis of chondrocytes from different embryonic origins was performed on pseudobulk counts using the R package muscat.
(3) scRNA-seq Data Integration Across Embryonic Origins
We filtered out potential doublets using the R package doubletFinder and removed clusters enriched for mitochondrial counts. The resulting UMI count matrix was divided by size factor and log-transformed using SCRAN. The top variable genes (getTopHVGs, SCRAN) identified in at least two samples were kept for downstream analyses. Using Seurat, we then integrated the count matrices using anchors in canonical correlation analysis (CCA) reduction, to compute batch corrected matrices of the three embryonic origins. To calculate co-embedding projections, the PCA dimension were reduced sample-wide. Anchors for integration were identified using 'FindIntegrationAnchors' in reciprocal PCA reductions. We used 'IntegrateEmbeddings' to integrate PCA reduction, followed by tSNE calculations ('RunTSNE'). Correlation analyses were performed on 'pseudobulk' average gene expression values (Seurat function 'AverageExpression') in each cluster.
(4) scRNA-seq Pseudotime Analyses
We generated spliced/unspliced count matrices of our selected mesenchymal populations using velocyto and assessed the directional transcriptional dynamics of highly variable genes with sufficient spliced/unspliced counts in scVelo with the default parameters. We visualized the recovered dynamics on tSNE projections of the three embryonic origins. We then used these tSNE embeddings as input space, and constructed a minimum spanning tree with preset start cluster in the R package slingshot. Alignment of embryonic origin-specific pseudotimes was performed with TrAGEDy using 40 interpolated points along the respective chondrogenic trajectories. Module expression dissimilarities were calculated by Spearman correlation (1-ρ) and optimal alignment was identified by dynamic time warping with default settings. Using the R package tradeSeq, we detected temporally differentially expressed genes along the respective chondrogenic trajectories.
(5) scATAC-seq Data Pre-Processing
We removed doublets with ArchR (v1.0.1) and selected high quality cells in Signac (v1.1.1) using the following thresholds: total number of fragments in peaks ranging from 1000 to 100000, fraction of reads in peaks >15%, nucleosome signal < 4 and TSS enrichment score > 2. Using these criteria, we ended up with 11527 cells for frontonasal (6171 and 5356 cells for HH15 and HH18), 14106 cells for somite (11232 and 2874 cells for HH12 and HH15), and 10982 cells for forelimb (4453 and 6529 cells for HH21 and HH24).
(6) scATAC-seq Data Merging, Dimensionality Reduction and Clustering
We merged samples from same embryonic origins and summed fragment counts in 5kb genomic tiling windows located on autosomes and chromosome Z (208680 tiles in total). We performed latent semantic indexing (LSI) dimension reduction on a term frequency-inverse document frequency (TF-IDF) normalized matrix with the top 75% of tiles (top 0.1% tiles are removed, putative repetitive elements or alignment errors) using Signac and removed batch effects on LSI components using Harmony. We used tSNE and FFT-accelerated Interpolation-based t-SNE algorithm to carry out non-linear dimensionality reduction with LSI dimensions 2:30, and performed Leiden graph-based clustering in Seurat on all cells with resolutions of 0.4 (nasal, somite) and 0.6 (limb) (='broad clustering'), and a second round of clustering on select mesenchyme populations with resolution of 0.4 (='fine clustering'). We annotated cell types for both 'broad' and 'fine' clusters using scATAC-seq gene activity matrices (promoters and gene bodies) and scRNA-seq expression data, with a combination of label transfers in Seurat and non-negative least squares (NNLS) regression on cluster specific genes, as well as manual inspection of peaks at known marker genes.
(7) Peak Calling and Differential Accessibility Analysis
We identified peaks using MACS2 (version 2.2.7.1) with parameters "--nomodel --shift 100 --extsize 200 --keep-dup all --call-summits" on pseudobulks of each cluster, for each embryonic origin, respectively. Peaks used summits as center and were extended to a width of 501bp. We merged peaks from different clusters of the same embryonic origin and, for overlapping peaks, kept only the most significant one, using adapted code from ArchR. To get a consensus peak set, we merged peaks from three embryonic origins and removed redundant and/or overlapping peaks using the same logic.
Differential accessibility analysis was performed in Seurat, using the total number of fragments and embryonic stages as latent variables. Peaks accessed in at least 10% of the cells and showing differences with adjusted p-value less than 0.05 and a log fold change larger than 0.25 were considered as significantly differentially accessed. Peak-centered heatmaps of differential accessible peaks were visualized with deepTools2.
(8) scATAC-seq Data Integration Across Embryonic Origins
The top variable peaks (getTopHVGs, SCRAN) identified in at least two samples were kept for downstream analyses. To calculate a co-embedding projection, first we performed reciprocal LSI dimensional reduction to find anchors (Seurat function 'FindIntegrationAnchors') and constructed transformation matrices between each query cell and anchor. We computed the integration matrices based on the original LSI matrix with dimensions from 2 to 30 and the transformation matrix using the Seurat functions 'IntegrateEmbeddings' and 'runTSNE' on the integrated LSI dimensions. To remove the batch effects among peak matrices after merging, we binarized the matrix based on presence/absence of counts. Correlation analyses were performed on 'pseudobulk' average count values in each cluster using the function 'AverageExpression'.
(9) scATAC-seq Pseudotime Analyses
We transferred pseudotime values from our scRNA analyses using the Seurat function 'TransferData'. First, we integrated scRNA-seq expression matrices and scATAC-seq gene activity matrices and performed CCA dimensional reduction to find anchors. We then constructed a transformation matrix between each query cell and each ancho (Seurat function 'FindTransferAnchors') and computed the transferred scATAC-seq pseudotimes based on the original scRNA-seq pseudotimes and the transformation matrices. For all three embryonic origins, we restricted this transfer to only chondrogenesis related cell type clusters.
(10) De Novo Motif Enrichment Analysis and Annotation
We performed de novo motif enrichment analysis for each cluster, using Homer 'findMotifsGenome.pl' with -mset vertebrates -size -250,250 -fdr 5 and motif length between 8-22 bp (Homer p-value < 1e-11), using highly accessible peaks for each cluster. We obtained candidate TF annotations for this set of de novo motifs with the help of three databases (Homer vertebrates, JASPAR20 vertebrates, CisBP v2 chicken) using Homer and STAMP. We selected the best matches based on scRNA-seq expression levels of the predicted TFs, and Spearman correlations between motif activity and gene expression of candidate TFs in scATAC and scRNA aggregates (default k=50, n=400; for small clusters, k=20, n=200). For motifs with similarity scores >0.8, only the one with the lowest p-value was retained. Additionally, we checked for paralog TFs expression along our pseudotime trajectories and calculated its expression correlation to motif activity. We combined annotated de novo motifs from each embryonic origin and calculated motif similarity scores using PWMEnrich. For our final set of annotated de novo motifs, we computed per-cell motif deviation scores using chromVAR and conducted analysis of differential motif activity using Seurat.
(11) RIME Analysis
Quantitative proteomics data was exported from FragPipe and analyzed using the MSstats R package v.4.13.0. Data was imputed using "AFT model-based imputation" and p-values and q-values for pairwise comparisons were calculated using the limma package.
(12) Peak-to-Gene Link Analysis
We generated imputed pseudoexpression data for each scATAC cell based on scRNA-seq data, using the ArchR function 'addGeneIntegrationMatrix'. 500 cell aggregates were generated with, with 100 cells per aggregate. We then computed the Pearson

correlation between peak accessibility and pseudoexpression in mesenchymal aggregates using the ArchR function 'addPeak2GeneLinks'. Clustering of peak-to-gene links was calculated by the hkmeans method in factoextra. Functional enrichment analyses of peak-to-gene link clusters was conducted in rGREAT.
(13) Evolutionary Conservation Analysis
We investigated sequence evolutionary conservation of CREs identified through peak-to-gene link analysis using the phastCons program. Specifically, we retrieved phastCons scores calculated based on multiple alignments of 77 vertebrate species, including 55 birds, from the UCSC Genome Browser website. For each position along the chicken genome, the phastCons score represents the probability of negative selection. We then calculated the average phastCons scores along the coordinates of CREs that are common between all three origins, CREs shared between two or more origins, and in origin-specific CREs.

For manuscripts utilizing custom algorithms or software that are central to the research but not yet described in published literature, software must be made available to editors and reviewers. We strongly encourage code deposition in a community repository (e.g. GitHub). See the Nature Portfolio guidelines for submitting code & software for further information.

Data

Policy information about availability of data

All manuscripts must include a data availability statement. This statement should provide the following information, where applicable:

- Accession codes, unique identifiers, or web links for publicly available datasets
- A description of any restrictions on data availability
- For clinical datasets or third party data, please ensure that the statement adheres to our policy

The functional genomics data generated in this study have been deposited in the GEO repository under accession codes GSE281769 [https://www.ncbi.nlm.nih.gov/geo/query/acc.cgi?acc=GSE281769] (scRNA-seq) and GSE281763 [https://www.ncbi.nlm.nih.gov/geo/query/acc.cgi?acc=GSE281763] (scATAC-seq)). Previously published samples (limb scRNA-seq stages HH21, 25 and 2777) are also available at GEO (accession code: GSE174565 [https://www.ncbi.nlm.nih.gov/geo/query/acc.cgi?acc=GSE174565]). The proteomics data generated in this study have been deposited to the ProteomeXchange Consortium with identifier PXD057934 [https://proteomecentral.proteomexchange.org/cgi/GetDataset?ID=PXD057934] via the MassIVE partner repository with MassIVE data set identifier MSV000096424 [https://massive.ucsd.edu/ProteoSAFe/dataset.jsp?task=5f4ce522448743eeae07ac7ff4064d5b].

Research involving human participants, their data, or biological material

Policy information about studies with human participants or human data. See also policy information about sex, gender (identity/presentation), and sexual orientation and race, ethnicity and racism.

Reporting on sex and gender	*Use the terms sex (biological attribute) and gender (shaped by social and cultural circumstances) carefully in order to avoid confusing both terms. Indicate if findings apply to only one sex or gender; describe whether sex and gender were considered in study design; whether sex and/or gender was determined based on self-reporting or assigned and methods used. Provide in the source data disaggregated sex and gender data, where this information has been collected, and if consent has been obtained for sharing of individual-level data; provide overall numbers in this Reporting Summary. Please state if this information has not been collected. Report sex- and gender-based analyses where performed, justify reasons for lack of sex- and gender-based analysis.*
Reporting on race, ethnicity, or other socially relevant groupings	*Please specify the socially constructed or socially relevant categorization variable(s) used in your manuscript and explain why they were used. Please note that such variables should not be used as proxies for other socially constructed/relevant variables (for example, race or ethnicity should not be used as a proxy for socioeconomic status). Provide clear definitions of the relevant terms used, how they were provided (by the participants/respondents, the researchers, or third parties), and the method(s) used to classify people into the different categories (e.g. self-report, census or administrative data, social media data, etc.) Please provide details about how you controlled for confounding variables in your analyses.*
Population characteristics	*Describe the covariate-relevant population characteristics of the human research participants (e.g. age, genotypic information, past and current diagnosis and treatment categories). If you filled out the behavioural & social sciences study design questions and have nothing to add here, write "See above."*
Recruitment	*Describe how participants were recruited. Outline any potential self-selection bias or other biases that may be present and how these are likely to impact results.*
Ethics oversight	*Identify the organization(s) that approved the study protocol.*

Note that full information on the approval of the study protocol must also be provided in the manuscript.

Field-specific reporting

Please select the one below that is the best fit for your research. If you are not sure, read the appropriate sections before making your selection.

☒ Life sciences ☐ Behavioural & social sciences ☐ Ecological, evolutionary & environmental sciences

For a reference copy of the document with all sections, see nature.com/documents/nr-reporting-summary-flat.pdf

Life sciences study design

All studies must disclose on these points even when the disclosure is negative.

Sample size	For scRNA-seq experiments, three independent samples per anatomical location were taken, at different embryonic stages, but capturing overlapping cell type repertoires (see Supplementary Figure 2). For scATAC-seq experiments, two independent samples per anatomical location were taken, at different embryonic stages, but capturing overlapping cell type repertoires. All tissue samples were taken from multiple pooled embryos at the same stage. For RIME experiments, four independent samples per anatomical location were taken, at the same embryonic stage. All tissue samples were taken from multiple pooled embryos.
Data exclusions	Low quality cells were excluded from further analyses, based on quality control metrics detailed in Supplementary Figure 1 (scRNA-seq) and Supplementary Figure 3 (scATAC-seq), see also Methods section for cut-off used.
Replication	For scRNA-seq and scATAC-seq experiments, cellular profiles were replicated within each individual sample, and 'pseudo-replicated' across samples, due to the overlapping cell type repertoires. For RIME experiments, four independent replicates were generated for each anatomical location and antibody used. For enhancer reporter experiments, at least five independent embryos needed to show GFP signal to be scored as 'positive'.
Randomization	No randomization was performed, as all samples needed to be correctly assigned to the correct tissue type and sampling stage, for proper interpretation of the results.
Blinding	No blinding was performed, as all samples needed to be correctly assigned to the correct tissue type and sampling stage, for proper interpretation of the results.

Reporting for specific materials, systems and methods

We require information from authors about some types of materials, experimental systems and methods used in many studies. Here, indicate whether each material, system or method listed is relevant to your study. If you are not sure if a list item applies to your research, read the appropriate section before selecting a response.

Materials & experimental systems

n/a	Involved in the study	
☐	☒	Antibodies
☒	☐	Eukaryotic cell lines
☒	☐	Palaeontology and archaeology
☐	☒	Animals and other organisms
☒	☐	Clinical data
☒	☐	Dual use research of concern
☒	☐	Plants

Methods

n/a	Involved in the study	
☒	☐	ChIP-seq
☒	☐	Flow cytometry
☒	☐	MRI-based neuroimaging

Antibodies

Antibodies used	For immunohistochemistry, antibodies against green fluorescent protein (GFP, Abcam, ab13970C46, chicken, 1:2000), tdTomato (tdT, Origene/Labforce, AB8181-200, goat, 1:1000) and SOX9 (MilliporeB101, AB5535C101, rabbit, 1:1000) were used, with corresponding secondary, fluorescently labeled antibodies from Jackson ImmunoResearch (1:500). For RIME experiments, antibodies against SOX9 (MilliporeB101, AB5535C101, rabbit, 5ug/replicate) or FOXP1 (Abcam, ab16645, rabbit, 5ug/replicate) were used.
Validation	For GFP and tdTomato, un-electroporated control embryos were used as negative staining controls. For SOX9 and FOXP1, a comparative RIME test run was performed to assess the specificity of the antibodies (see Supplementary Figure 7a-d)

Animals and other research organisms

Policy information about studies involving animals; ARRIVE guidelines recommended for reporting animal research, and Sex and Gender in Research

Laboratory animals	Fertilized chicken eggs (Gallus gallus domesticus, "Hubbard") were purchased from local vendors in Switzerland and incubated to the desired stages in a humidified incubator.
Wild animals	No wild animals were used in this study.
Reporting on sex	Embryos were not sexed prior to tissue collection.
Field-collected samples	No field-collected samples were used in this study.

Ethics oversight

In accordance with Swiss national guidelines (Swiss Animal Protection Ordinance; TSchV, chapter 6, Art. 112), no formal ethics approval was required, as all experiments were carried out prior to the third trimester of incubation.

Note that full information on the approval of the study protocol must also be provided in the manuscript.

Plants

Seed stocks

Report on the source of all seed stocks or other plant material used. If applicable, state the seed stock centre and catalogue number. If plant specimens were collected from the field, describe the collection location, date and sampling procedures.

Novel plant genotypes

Describe the methods by which all novel plant genotypes were produced. This includes those generated by transgenic approaches, gene editing, chemical/radiation-based mutagenesis and hybridization. For transgenic lines, describe the transformation method, the number of independent lines analyzed and the generation upon which experiments were performed. For gene-edited lines, describe the editor used, the endogenous sequence targeted for editing, the targeting guide RNA sequence (if applicable) and how the editor was applied.

Authentication

Describe any authentication procedures for each seed stock used or novel genotype generated. Describe any experiments used to assess the effect of a mutation and, where applicable, how potential secondary effects (e.g. second site T-DNA insertions, mosiacism, off-target gene editing) were examined.
